# Circulating genotypes of *Leptospira* in French Polynesia : An 9-year molecular epidemiology surveillance follow-up study

**DOI:** 10.1371/journal.pntd.0008662

**Published:** 2020-09-28

**Authors:** Linda Grillová, Hilde Angermeier, Marc Levy, Marine Giard, Stéphane Lastère, Mathieu Picardeau

**Affiliations:** 1 Institut Pasteur, Biology of Spirochetes Unit, Paris, France; 2 European Programme for Public Health Microbiology (EUPHEM), European Centre for Disease Prevention and Control, (ECDC), Stockholm, Sweden; 3 Centre hospitalier de Polynésie française, Papeete, French Polynesia; 4 Bureau de veille sanitaire, Direction de la Santé, Papeete, French Polynesia; Yale University School of Medicine, UNITED STATES

## Abstract

**Background:**

Leptospirosis is a widespread zoonosis with global impact, particularly among vulnerable populations in resource-poor settings in tropical countries. Rodents have been considered to be the main reservoir of the disease; however, a wide variety of mammals can act as hosts as well. Here we examine the genetic diversity of *Leptospira* strains from biological samples of patients and animals in French Polynesia (FP) from 2011 to 2019.

**Methodology/Principal findings:**

From 2011 to 2019, we have collected 444 blood samples from patients diagnosed as having leptospirosis. The limited volume of clinical material and low amount of leptospiral DNA in blood samples led us to develop a nested PCR targeting the *secY* locus that enabled us to amplify and sequence 244 samples (55%). In addition, 20 *Leptospira* strains recovered from the blood of patients from 2002 to 2011 were sequenced and fully characterized at the serogroup level and used as reference strains for the association of different phylogenetic branches with respective serogroups. The *secY* sequences were compared with publicly available sequences from patients and animal reservoirs in FP (n = 79). We identified rats as the main source of infection for *L*. *borgpetersenii* serogroup Ballum and *L*. *interrogans* serogroup Icterohaemorrhagiae, dogs as the main source of infection for *L*. *interrogans* serogroup Australis, and farm pigs as the main source of infection for *L*. *interrogans* serogroups Pomona or Canicola. *L*. *interrogans* was associated with the most severe infections with 10 and 5 fatal cases due to serogroups Icterohaemorrhagiae and Australis, respectively. Mortality was significantly associated with older age (p-value < 0.001).

**Conclusions/Significance:**

We described the population dynamics of leptospires circulating among patients in FP, including two patients who were reinfected with unrelated *Leptospira* genotypes, and clarified the local role of the animal reservoirs in the transmission route of leptospirosis to humans. Routine *Leptospira* genotyping directly on biological samples should allow the epidemiological follow-up of circulating strains and assess the impact of control interventions on disease transmission.

## Author summary

Pathogenic *Leptospira* are the causative agents for leptospirosis, a neglected zoonosis occurring worldwide with the highest incidence of the disease found in the Pacific region, including French Polynesia (FP). In this study, a nested PCR of blood specimens allowed a high isolation frequency of *Leptospira* DNA among patients with febrile illness in FP. Infecting *Leptospira* isolates were characterized by sequencing of the *secY* gene from 244 clinical samples from 2011 to 2019. The genotypes have been assigned to *Leptospira* species and serogroups by comparing the *secY* sequences with the genomes of strains isolated prior to 2011 in FP. This study therefore enables to follow the distribution of genotypes/serogroups over a 18-year time period. Leptospiral sequences from patients were also compared with *secY* sequences from different animal reservoirs from a previous study in FP showing that not only rats but also dogs and pigs might play an important role in the circulation of pathogenic *Leptospira* strains in FP. Further progress in our understanding of the epidemiology of *Leptospira* circulating genotypes should contribute to the implementation of prevention and intervention measures to reduce the risk of leptospirosis transmission in FP.

## Introduction

Leptospirosis, an emerging zoonotic disease, is caused by spirochetes of the genus *Leptospira*. It comprises 64 species that fall into phylogenetic clusters correlating with virulence—the saprophytes (S1, S2), the intermediates (P2) and the pathogens (P1) [1]. Annually, pathogenic *Leptospira* cause more than one million severe cases and 60,000 deaths worldwide [2]. However, leptospirosis is under-diagnosed because of nonspecific clinical manifestations and poor sensitivity of current diagnostic tests. Rats are asymptomatic carriers and serve as the main reservoir, but *Leptospira* spp. can colonize the renal tubules of a wide variety of wild and domesticated mammals. The bacteria are shed in the urine of infected animals and persist in freshwater [3]. Transmission to a new host usually occurs after exposure to this contaminated water [4]. Infections are more common in low-resource, tropical and subtropical locations and outbreaks often occur after natural disasters such as hurricanes with increased rainfall and flooding [5].

This study focuses on French Polynesia (FP) which is a French Overseas Country consisting of 22 territories in the South Pacific Ocean encompassing 118 atolls and islands [6]. Its overall population has been estimated to be 270,000 inhabitants. Tahiti is the most populated island of FP with around 70% of its population living there. FP is a hotspot for leptospirosis with an annual incidence of 30 to 55 cases per 100,000 inhabitants [7]. Bouscaren and colleagues reported the surveillance data of 1,356 confirmed and probable cases of leptospirosis from 2007 to 2017 showing that the prevalence of leptospirosis is much higher in males and that farming and breeding activities as well as contact with animals are potential risk factors [8]. Leptospirosis in FP is also associated with heavy rainfalls and occurs predominantly in the high islands (Leeward Islands, Windward Islands and Marquesas). Rats were identified as a major source for human leptospirosis in FP by genotyping *Leptospira* DNA from patients (n = 33) and different animal reservoirs including rats, farm pigs and domestic dogs [7]. However, the epidemiological data are still incomplete and many questions remain unanswered: What is the role of farm pigs in the transmission of leptospirosis in FP [7], what are the strains associated with disease severity, and do we see the emergence of new strains during long-term follow up?

We have sequenced and analyzed 264 samples collected from patients having leptospirosis during 2002–2019 in FP with the following objectives: i.) clarify the role of the animal reservoir for pathogenic *Leptospira* species and address the transmission route to humans, ii.) describe the molecular epidemiology of leptospirosis and identify the trends in population dynamics of *Leptospira* genotypes over the time, and iii.) determine, if some strains were associated with severe evolution of the disease. This study shows that molecular epidemiological surveillance of leptospirosis can be performed by direct genotyping from biological samples. These findings should contribute to establishing appropriate control and prevention measures to reduce the burden of this emerging disease.

## Methods

### Collection of clinical samples, isolates and serotyping

From 2011 to 2019, we have collected 444 serum samples from patients diagnosed with leptospirosis at the Centre hospitalier de Polynésie Française in Tahiti, FP. The diagnosis was based on the clinical symptoms in combination with qPCR screening. The DNA was isolated using the Magna Pure LC DNA Isolation kit (Roche, France). DNA samples were then sent to the National Reference Center for Leptospirosis (NRC) at the Institut Pasteur in Paris, France, for further molecular analysis. In addition, 20 *Leptospira* strains isolated from patients from 2002 and 2011 were used as reference strains. *Leptospira* strains were grown in EMJH liquid medium at 30°C. Species identification was performed by amplification and sequencing of the 16S rDNA. Serological characterization of isolates was performed at the National Reference Center for *Leptospira* by using rabbit antisera against reference serovars representing a standard battery of 24 serogroups [9].

### Molecular typing

The conventional PCR for amplifying *secY* was performed as described previously [10]. Nested PCR was performed using the newly designed primers (S1 Table) amplifying a 410 bp-long DNA fragment under the following conditions: 95°C (1 min); 94°C (30 s), 55°C (30 s), and 72°C (1 min) for 45 cycles; with the final extension at 72°C for 10 min. The second step of nested PCR was performed under the same conditions, but with a decreased annealing temperature (52°C). Each PCR mixture contained 1U of GE Healthcare Recombinant Taq DNA Polymerase (Scientific Laboratory Supplies, Nottingham, UK), 5μM of dNTP, 25mM of MgCl_2_, 5 μl of 10x buffer, 10μM of each primer, 5 μl of DNA obtained from patient serum samples in the first step, and 1 μl of the completed initial PCR reaction in the second step. The reaction mixture was supplemented with PCR-grade water to a final volume of 50 μl.

### DNA sequencing and data availability

Sanger sequencing of PCR products was performed by Eurofins Genomics Germany GmbH (Ebersberg, Germany) and the sequence analyses were performed with Lasergene software (DNASTAR v. 7.1.0.; DNASTAR, Madison, WI). NGS was performed for strains 200800517, 200212385, as well as 20040363 using Nextera XT DNA Library Preparation kit and the NextSeq 500 sequencing systems (Illumina, San Diego, CA, USA) at the Mutualized Platform for Microbiology (P2M) at Institut Pasteur. The data were analyzed using CLC Genomics Workbench 9 software (Qiagen, Hilden, Germany). Partial *secY* sequences representing all genotypes found in this study (genotype 1 –genotype 20) were deposited in GenBank under the following accession numbers: MT235218—MT235235. The whole genome sequences of the selected strains were submitted to the publicly available BIGSdb hosted at Institut Pasteur MLST and whole genome MLST databases (Bacterial Isolate Genome Sequence database, https://bigsdb.pasteur.fr/leptospira/).

### Phylogenetic analyses and statistics

Maximum likelihood phylogenetic trees were generated with MEGA 6 [11] using the Tamura Nei model and 1000 pseudorandom bootstrap replicates. Median-joining networks were generated with Network version 4 [12]. Deletions and insertions were counted as single events. The population dynamic of identified genotypes was shown by Muller diagram generated in R-studio (version 3.6.1.) using the MullerPlot package [13]. The statistical interference was calculated in R-studio (version 3.6.1). We have used t-tests or ANOVAs for numerical variables (age, CT-values) and comparison of proportions and chi-square tests of independence for categorical variables (sex of the patients, clinical outcome, year/season of sampling, *Leptospira* species, lineages, genotypes and serogroups). The level of significance was settled to 0.05.

### Ethics statement

A written informed consent from patients was not required as the study was conducted as part of routine surveillance of the Centre Hospitalier de Polynésie française, French Polynesia, and no additional clinical specimens were collected for the purpose of the study. Human samples were anonymized. Collection of the strains was conducted according to the Declaration of Helsinki.

## Results

### Design and discrimination power of nested PCR based on *secY* gene

We extracted *secY* gene sequences from 707 genomes of pathogenic *Leptospira* available at the Institut Pasteur MLST database (https://bigsdb.pasteur.fr/leptospira/). This sample set represents all pathogenic strains that had been sequenced at the time of the design (June 2019) isolated from different hosts across the globe. The primers for nested PCR (S1 Table) were designed based on sequence alignment to amplify the highly variable region of *secY*. The *secY* outer primers were used for the amplification of the first PCR products (554 bp) and *secY* inner primers were used for the amplification of the second and final PCR products. The newly designed nested PCR targeted a 410 bp-long DNA fragment only. Even though the shortened fragment of 410 bp lost 10% of its discrimination power compared to the longer fragment (554 bp), it was still able to distinguish 52%, 56% and 53% of concatenated sequences of typing genes from the MLST 1 (3,111 bp-long concatemer of 7 loci) [14], MLST 2 (4,189 bp-long concatemer of 7 loci) [15] and MLST 3 (2,980 bp-long concatemer of 6 loci) [10], respectively.

Phylogenetic analyses of the partial 410-nt fragment *secY* sequences of 707 pathogenic *Leptospira* isolated across the globe (including 17 pathogenic species and 22 serogroups) revealed 114 genotypes, in comparison to 300 CGs when using the 545 core genes defined by cgMLST [16]. The *secY* sequences can discriminate strains at the species and subspecies levels but, like the MLST schemes, the level of discrimination does not always predict the serogroup.

### Molecular typing

From 2011 to 2019, we collected 444 blood samples from patients diagnosed as having leptospirosis in FP. Because of the low amount of *Leptospira* DNA present in the samples, it was not always possible to sequence *secY* in qPCR positive samples. A nested PCR was therefore designed to increase the amplification efficiency, and, due to the limited volume of clinical material available, was applied only to a subset of samples. The amplification efficiency using traditional and nested PCR reached 44% and 65%, respectively, making nested PCR the method of choice for future studies. A combination of traditional and nested PCR has enabled us to amplify and Sanger sequence the *secY* locus in 244 samples (55%). Among our sample set, the vast majority of patients were male (82%). The median age was 37 (8–88) years old. Patients originated from Tahiti (n = 177), Huahine (n = 21), Raiatea (n = 17), Moorea (n = 14), Bora Bora (n = 6), Tahaa (n = 4), Marquesas (n = 2), Gambier (n = 2), and Austral (n = 1) islands (S2 Table).

**Fig 1:**
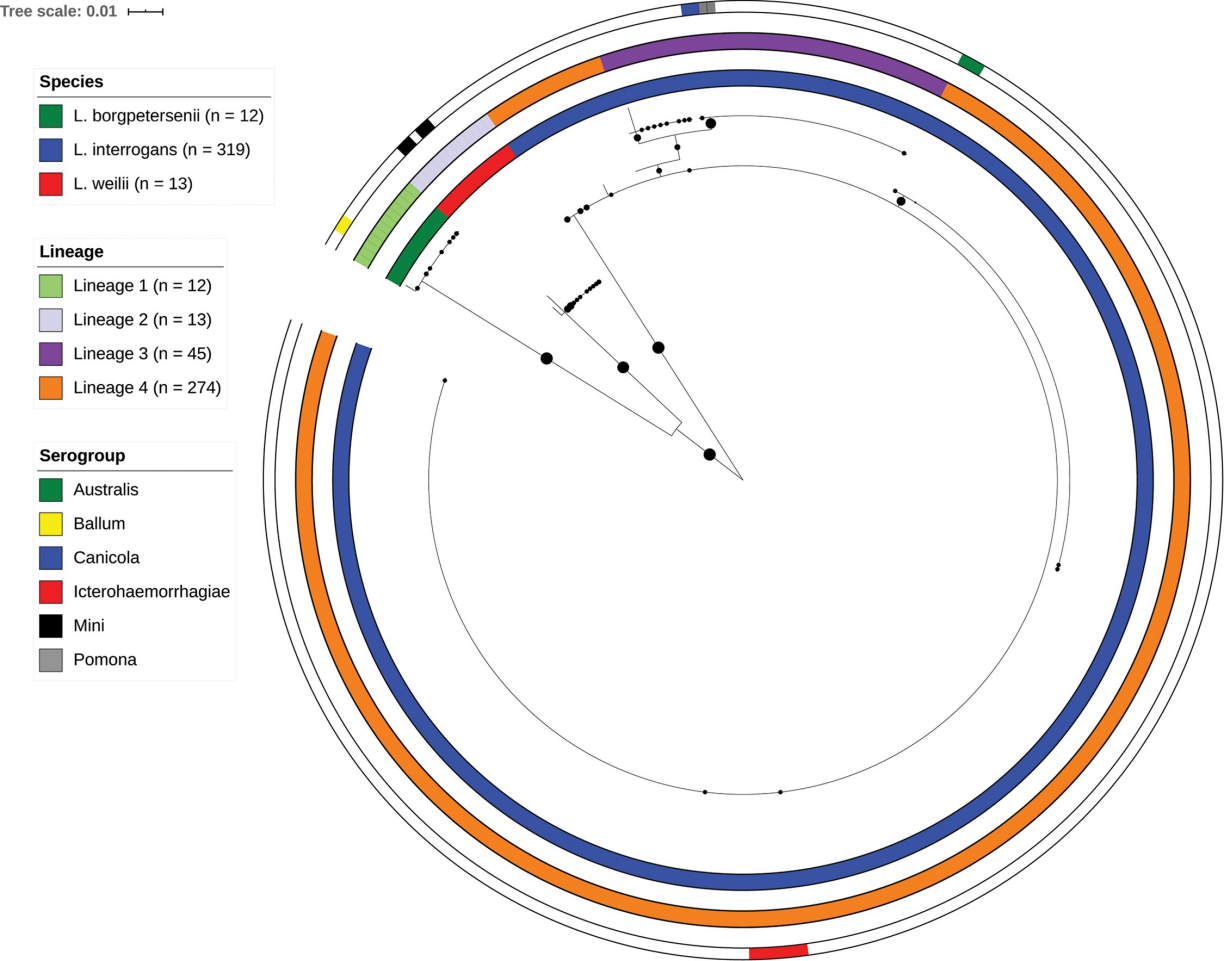
Maximum likelihood phylogeny (1000 bootstrap replicates) of *Leptospira sec*Y sequences from patients. The phylogeny shows the distribution of species lineages, and serogroups circulating in FP during the time span from 2002 to 2019 (n = 344). Bootstrap values are shown with the size of circles in the middle of the branches, bootstraps lower than 70 are not shown. Species, lineage and serogroup names are given by the color codes (from inner to outer circles). Serogrouping was performed on culture isolates. White color indicates that the information was not available.

**Fig 2:**
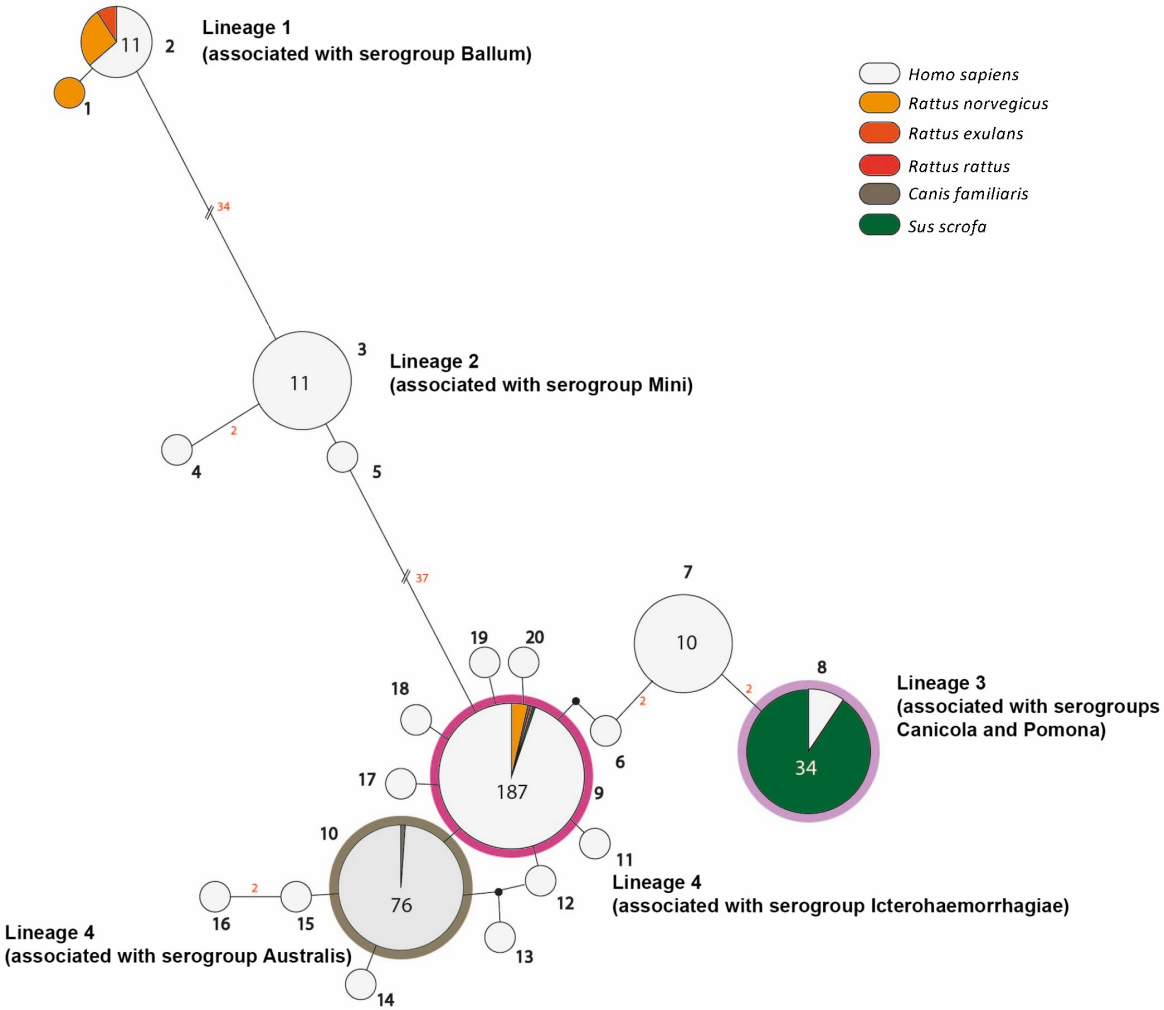
Median-joining network showing different *Leptospira* genotypes isolated from different hosts and the number of mutational differences among them. The number of mutations, when > 1, is given adjacent to branches (in red). Inferred allelic variants (median vectors) are shown as small black connecting circles. If contiguous, indels were considered as single events only. The numbers of individuals, when > 1, are shown inside circles and are indicated by circle size. The color code indicates the host. Genotypes (in bold) and lineages are shown adjacent to the associated haplotypes.

We have combined our data with *secY* data already available from a previous study in FP [7] that enhanced our sample set up to 321 samples (S2 Table). Samples were collected from human patients (n = 33) and from animal reservoirs (rats, n = 15; dogs, n = 2; and pigs, n = 29) from 2012 to 2015 [7]. The majority of the samples originated from Tahiti (71%) which is the largest and most populated island in FP [8]. In addition, 20 *Leptospira* strains isolated from patients in FP between 2002 and 2011 were included in our study. Serogrouping was performed on these cultured strains (Australis, n = 3; Ballum, n = 2; Canicola, n = 2; Icterohaemorrhagiae, n = 8; Pomona, n = 2; Mini, n = 4). These strains served as references for the association of different phylogenetic branches with respective serogroups. The analysis of the 410-bp *secY* fragment divided our sample set into 20 genotypes that clustered into 4 lineages (Fig 1, Fig 2). Every genotype differed by at least one single nucleotide variant (SNV) from the others. The lineages were assigned based on the phylogeny and differed from each other by at least 4 SNVs.

Lineage 1 contained 12 samples belonging to *L*. *borgpetersenii*. This lineage was shared between patients as well as rats and was associated with the serogroup Ballum. Lineage 2 contained 13 samples of *L*. *weilii*, was found exclusively in patients, and was associated with the serogroup Mini. The vast majority of samples were identified as *L*. *interrogans* creating 15 different genotypes that clustered into two lineages. Lineage 4 was represented by 12 genotypes with two central ones; genotype 10 (n = 75) found mostly in humans, but also in one dog and associated with serogroup Australis; and genotype 9 (n = 188). The latter was the most prevalent genotype, which is shared among humans, rats (n = 10), one dog, and one pig. It is associated with serogroup Icterohaemorrhagiae (Fig 2). Lineage 3 was divided into 3 genotypes. The genotype 8 was isolated from patients as well as from farm pigs (n = 28) (Fig 2) and was associated with both serogroup Canicola and serogroup Pomona. To further discriminate the two different serogroups sharing the same *secY* sequence, we performed whole genome sequencing and cgMLST based on 545 core genes [16]. The strains from serogroup Pomona (ids 200403463 and 200210107) belonged to cgMLST clonal group 5, while the strain from serogroup Canicola (id 200212385) belonged to cgMLST clonal group 28, confirming that the *secY* sequence alone cannot discriminate between these two serogroups.

When focusing on human leptospirosis, we found that most were males (n = 206, 83%) and that the age of the patients ranged from 8 to 88 years with a median of 33 and a mean of 37 (99% CI: 33.91–39.24) years. The statistical interference was calculated for average age and categorical variables such as sex, clinical outcome (death or recovery), *Leptospira* species, lineages and genotypes. Unsurprisingly, we found a statistically significant difference in the average age of patients who died of leptospirosis compared to those who survived (p-value < 0.001). The older patients (median age 56) had a higher rate of mortality than the younger patients (median age 35) (S1 Fig). In total, we have collected samples from 15 patients who died as a consequence of leptospirosis. All patients were infected with the strains belonging to lineage 4 (*L*. *interrogans* associated with serogroups Icterohaemorrhagiae, n = 10; and Australis, n = 5). Interestingly, we have identified a significant difference in the average age of patients who were infected by different lineages (S2 Fig). Compared to lineages 2, 3 and 4 infecting individuals with average age 33, 27, 37, respectively, lineage 1 (*L*. *borgpetersenii* associated with the serogroup Ballum) was more likely to infect patients with higher average age (57, p-value = 0.0268). Apart from this outcome, no other correlations have been found. During our follow-up, reinfections with unrelated genotypes was also reported for two patients. One individual was reinfected with a different Lineage 4 genotype (*L*. *interrogans* associated with serogroups Australis) 5 years after the initial infection (*L*. *interrogans* associated with serogroups Icterohaemorrhagiae). For the other individual, reinfection with an unrelated genotype (Lineage 2; *L*. *weilii*) occurs 1 year after the first infection (Lineage 3; *L*. *interrogans*). These observations suggest that a previous infection may not protect against a subsequent infection.

## Discussion

Pathogenic *Leptospira* strains are fastidious and slow-growing bacteria making it difficult to isolate the infecting strains from biological sources. Alternatively, genetic markers can be directly amplified from the samples and sequenced to assess the genetic diversity of the bacterium. A number of loci including *rrs* [9, 17], *ppk* [2], *ligB* [9], *lflb1* [18, 20], *secY* [7, 17–22] were used to identify the circulating *Leptospira* genotypes in biological samples. To facilitate a comparison with the previous studies in FP [7], we have amplified, sequenced and analyzed the *secY* gene from biological samples collected from patients from 2011 to 2019. *SecY* is a widely used target for phylogenetic analysis of *Leptospira* because of its presence in all *Leptospira* species and its high sequence diversity enabling discrimination at the subspecies level [22]. This gene has already been used in a molecular typing scheme (MLST3) [10] and as a single typing locus in many epidemiological studies [7, 17–22]. In addition, we have revealed that phylogeny based on the *secY* locus of available *Leptospira* strains in our database (n = 707) has a discrimination power equivalent to the 38%, 52%, 56% and 53% of phylogenies based on cgMLST, MLST 1, MLST 2 and MLST 3, respectively. Moreover, despite the high sequence diversity, the phylogeny based on the *secY* locus is able to follow the branching of phylogeny based on core genomes, thus enabling accurate identification of *Leptospira* strains at the species level. A single-locus target has been chosen for the following reasons: i.) usage of a limited volume of the according clinical sample and ii.) low amount of *Leptospira* DNA present in the serum samples; both preventing successful amplification of multi-locus targets for MLST. The lower sensitivity of PCR in serum samples compared to whole blood samples has already been observed [23, 24]. The development of a nested PCR enabled enhancement of the amplification efficiency [25]. This, in turn, enabled us to amplify and sequence 55% of the available serum samples of leptospirosis patients in our study.

All mammals can act as reservoirs for leptospirosis. Contact with livestock (cattle, horses and swine), dogs, rats and cats have been previously reported as potential risk factors of leptospirosis in FP between 2007 to 2017 [8] but studies investigating animal leptospirosis in FP are currently limited [7]. In addition to the study from Guernier *et al*. [7] where *Leptospira secY* sequences were amplified from pigs, rats, and dogs, a serological study has also shown that cattle and horses can be seropositive for leptospirosis [26]. There are no reliable estimates of populations of potential reservoirs in FP. Stray dogs are present in all the islands and pig farming by the rural population is an inherent element of ancestral Polynesian culture. Although the role of rats in leptospirosis is well established, there is increasing evidence that other domestic or wild animals may also serve as important hosts for pathogenic *Leptospira* [27]. Animal population surveys in FP, including their interactions with people, are needed to better understand their zoonotic disease transmission potential.

The *secY* sequenced from our clinical samples and the previously identified *secY* sequences from FP [7] cluster into four distinct lineages. At least one genotype from each lineage was found in human samples as well as in samples of animal origin enabling us to identify the source of infection. Lineage 2 (*L*. *weilii* associated with serogroup Mini) is the only exception, as it was found exclusively in human samples leading to the conclusion that the reservoir of this lineage remains unknown. Further ecological studies are needed to identify the reservoir host(s) of this genotype. We have identified rats as a potential source of infection for lineage 1 (*L*. *borgpetersenii*, associated with serogroup Ballum). Farm pigs were found as a potential source of infection for lineage 3. Based solely on the *secY* sequences obtained herein we were unable to identify if the infections were due to *L*. *interrogans* serogroup Canicola or *L*. *interrogans* serogroup Pomona. However, serovar Pomona has been the most common serovar isolated from pigs worldwide [28] suggesting that the strain infecting pigs in FP is associated with *L*. *interrogans* serogroup Pomona. Rats and dogs were identified as potential sources of infection with *L*. *interrogans* serogroup Icterohaemorrhagiae and *L*. *interrogans* serogroup Australis from lineage 4, respectively (Fig 2). In contrast to the previous study from 2017 [7], genotype 8 (belonging to lineage 3) was isolated from patients as well as from farm pigs. This suggests strongly that farm pigs represent a source of human infection in Tahiti.

*Leptospira* serovars or serogroups usually demonstrate specific host preferences [6]. Our findings are in agreement with our current knowledge of host preferences: i.e. the hosts for serogroups Icterohaemorragiae and Ballum are rodents, serogroup Australis can be found in dogs, whereas pigs are the reservoir for the serogroup Pomona (see above). In our study, the assignment of serogroups cannot rely exclusively on the *secY* sequence. Serogroup designation was attributed by comparing the *secY* sequences with those of 20 local strains belonging to 6 serogroups and representing the diversity of strains circulating in FP. However, strains of serogroups Canicola and Pomona belonging to genotype 8 share the same *secY* sequence and are indistinguishable. In contrast, cgMLST based on the 545 core genes can discriminate the strains that belong to the serogroups Canicola and Pomona.

**Fig 3:**
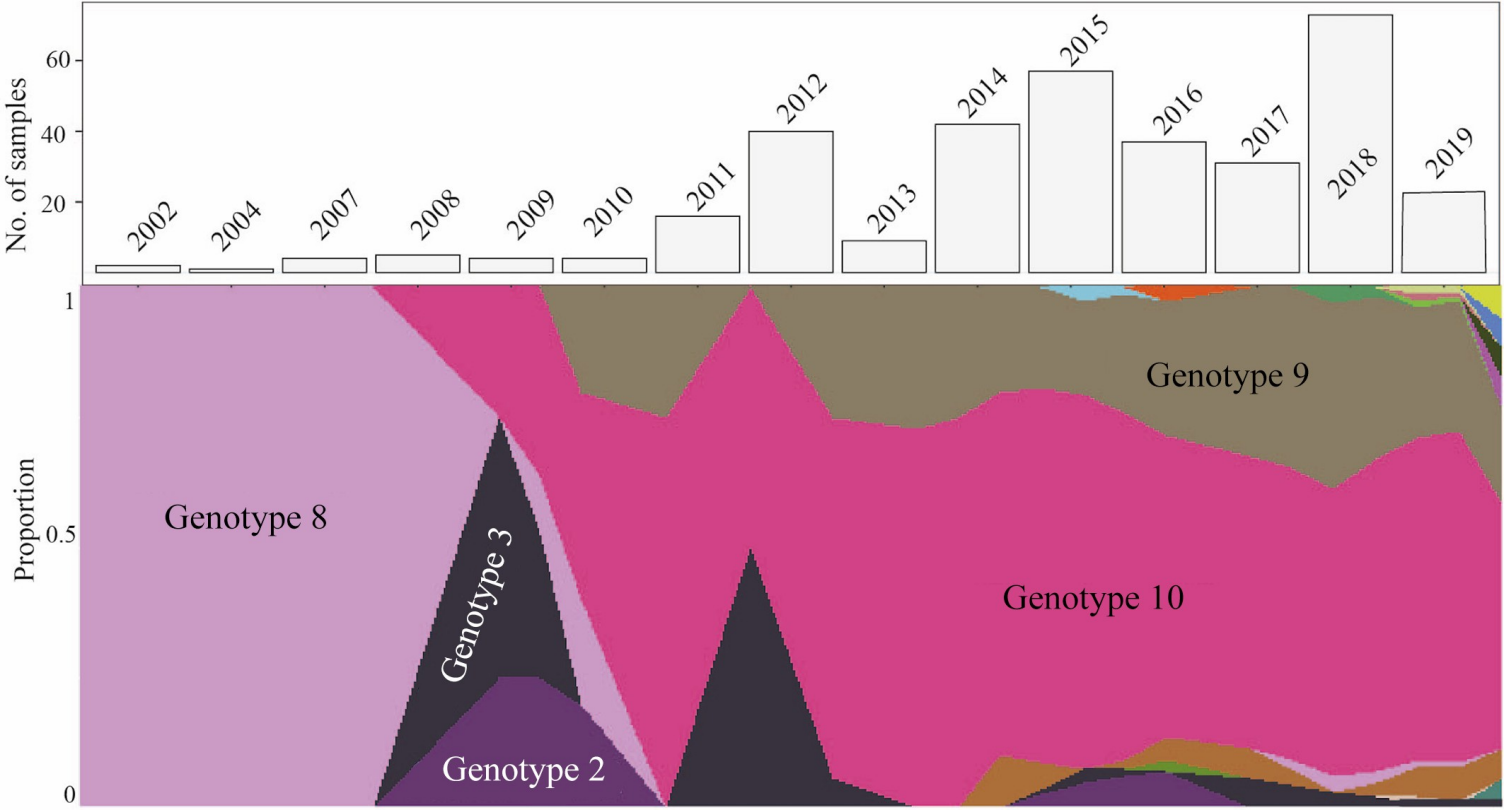
Muller diagram—Proportions of genotypes infecting human hosts in Tahiti during the 18-year sampling framework (lower panel). The most prevalent genotypes are highlighted. The bar chart (upper panel) is showing the number of samples sequenced in each year.

We did not observe major changes in the distribution of genotypes during the period 2011–2019 (n = 322). Examining the trend of the genotype distribution before 2011 is not possible due to the small sample size (n = 20 during the period 2002–2010). The most prevalent lineage 4 (*L*. *interrogans*) consisted of two main central genotypes, genotype 9 (associated with serogroup Icterohaemorrhagiae) and genotype 10 (associated with serogroup Australis). Genotype 9, which has been detected over a 10-years time span (since 2008), represented the predominant genotype found in FP (Fig 3). Several other studies have shown that predominant leptospiral strains in a particular population can change over time. This can be explained by a change in the relative populations of maintenance hosts [29]. Genotype 9 has been isolated from multiple animal reservoir hosts (including rats, dogs and pigs), which is not true of the other genotypes. The ability of genotype 9 to colonize a wide range of hosts and to cause severe infections (see below) can be the reason for its high prevalence and predominance over the 18-year time period.

The fact that most of the patients in our sample set (83%) were males corresponds well with the findings of many other epidemiological studies, where males were found to be the major victims of this pathogen [3, 6]. In addition, leptospirosis is known to be seasonal, and strongly associated with rainfalls in tropical settings. However, we did not observe any correlation between species, lineages or genotypes and the season in which the samples were collected.

We have found that older patients (median age 56) tend to die more frequently as a consequence of leptospirosis than younger ones (median age 35), suggesting that the immunity and the general physical shape of the patients play an important role in the manifestation of this disease. All dead patients were infected by leptospires belonging to lineage 4 (*L*. *interrogans* associated with serogroup Australis and Icterohaemorrhagiae), however, no correlation between lineages and the outcome of the disease was observed, since leptospires from lineage 4 were actually the most prevalent ones in our sample set (infecting 81% of examined individuals). However, in contrast to the leptospires belonging to the serogroup Icterohaemorrhagiae that were already associated with disease severity [30–32], we also identified leptospires belonging to the serogroup Australis as a causative agent of fatal outcome. In addition, *L*. *borgpetersenii* associated with serogroup Ballum (lineage 1) have been found more likely to infect older patients (p-value = 0.0268). Therefore, future studies should be designed to better identify the risk factors associated with disease severity such as clinical (e.g. bacteremia) and biological findings recorded at admission as well as social risk factors (e.g. occupational activity). Our study also shows that a first infection may not contribute to protect against a subsequent reinfection. Thus, reinfection with an unrelated genotype was responsible for severe diseases in two patients. In one case, reinfection occurred as early as 1 year showing that the first infection has not conferred crossprotection among an unrelated *Leptospira* species (*L*. *interrogans* vs *L*. *weilii*). Naturally-acquired immunity to reinfection in humans is poorly documented [33, 34] and further investigations are needed to better understand if some individuals are at risk of repeated infections. Such study may also have major implications for the development of an effective vaccine for leptospirosis in both humans and animals. Today, the only vaccines available against leptospirosis consist of killed whole-cell bacterins. These *Leptospira*-based vaccines have a narrow range of specificity and provide only short-term immunity (6).

In conclusion, using a sensitive nested PCR assay that can discriminate strains at the subspecies level, we identified 20 *Leptospira* genotypes circulating in both, humans and animals, in FP. This tool contributes to identify new animal reservoirs for leptospires and thus facilitates the early detection of emerging new genotypes. Our results emphasize the need for appropriate public health interventions to control not only rodents but also dogs and pigs as sources of these strains that are causing life-threatening infections.

## Supporting information

S1 Fig Side-by-side boxplots of the age of patients who died as a consequence of leptospirosis and those who survived. Mortality was significantly associated with older age (p-value < 0.001).
(TIFF)

S2 Fig Side-by-side boxplots of the age of patients infected with different phylogroups of *Leptospira*. Compared to lineages 2, 3 and 4, lineage 1 (*L*. *borgpetersenii* associated with the serogroup Ballum) was significantly more likely to infect patients with higher average age (57, p-value = 0.0268).
(TIFF)

S1 Table Primers used for nested PCR amplification of *secY* locus. (DOCX)

S2 Table Clinical samples examined in this study. (XLSX)
