# Supplementary figures and images for: Circulating genotypes of Leptospira in French Polynesia : An 9-year molecular epidemiology surveillance follow-up study
Source: PLoS Negl Trop Dis. 2020 Sep 28;14(9):e0008662. doi: 10.1371/journal.pntd.0008662 (PMC7544043; doi:10.1371/journal.pntd.0008662)

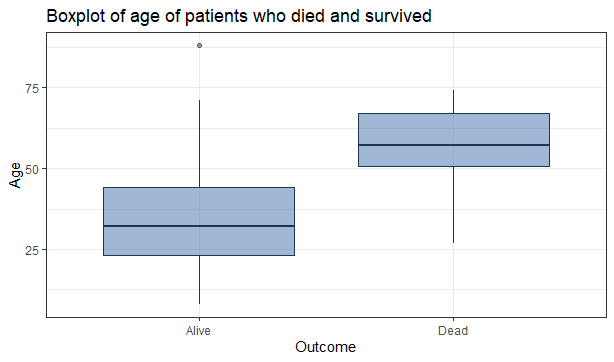

Supplement: S1 Fig — Mortality was significantly associated with older age (p-value < 0.001). (TIFF) [file pntd.0008662.s001.tiff]

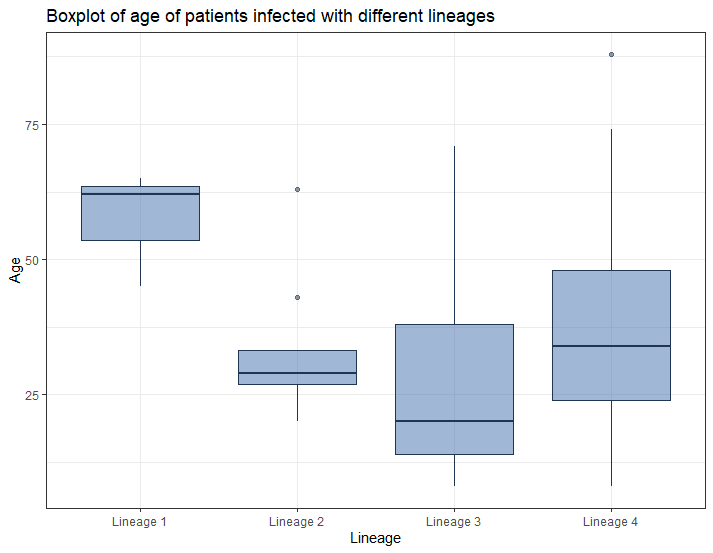

Supplement: S2 Fig — Compared to lineages 2, 3 and 4, lineage 1 (L. borgpetersenii associated with the serogroup Ballum) was significantly more likely to infect patients with higher average age (57, p-value = 0.0268). (TIFF) [file pntd.0008662.s002.tiff]
