# Supplementary material for: Circulating genotypes of Leptospira in French Polynesia : An 9-year molecular epidemiology surveillance follow-up study
Source: PLoS Negl Trop Dis. 2020 Sep 28;14(9):e0008662. doi: 10.1371/journal.pntd.0008662 (PMC7544043; doi:10.1371/journal.pntd.0008662)
Supplement: S1 Table — (DOCX) [file pntd.0008662.s003.docx]

**S1 Table: Primers used for nested PCR amplification of *secY* locus**

| Primer ID | Primers (5-3) | Length of the PCR product (bp) | Reference |
| --- | --- | --- | --- |
| secY_outer_F | ATGCCGATCATTTTTGCTTC | 554 | Ahmed et al., 2006 |
| secY_outer_R | CCGTCCCTTAATTTTAGACTTCTTC |  |  |
| secY_inner_F | CCTCAGACGATTATTCAATGGTTATC | 410 | This study |
| secY_inner_R | AGAAGAGAAGTTCCACCGAATG |  |  |
